# The Moderating Effect of COVID-19 Risk Perception on the Relationship Between Empathy and COVID-19 Volunteer Behavior: A Cross-Sectional Study in Jiangsu, China

**DOI:** 10.3389/fpubh.2022.863613

**Published:** 2022-06-16

**Authors:** Yeyang Zhu, Jie Zhuang, Baohua Liu, Huan Liu, Jiaojiao Ren, Miaomiao Zhao

**Affiliations:** ^1^Department of Health Management, School of Public Health, Nantong University, Nantong, China; ^2^Department of Law, School of Economics and Management, Nantong University, Nantong, China; ^3^School of Health Services and Management, Ningbo College of Health Sciences, Ningbo, China; ^4^Department of Social Medicine, Health Management College, Harbin Medical University, Harbin, China; ^5^Department of Preventive Medicine, Zhuhai Campus of Zunyi Medical University, Zhuhai, China

**Keywords:** empathy, COVID-19 risk perception, COVID-19 volunteer behavior, public health emergencies, moderating analysis

## Abstract

The health system has encountered great challenges since the COVID-19 outbreak, volunteers are urgently needed in every situation during this crisis. The current study aimed to explore the relationship between empathy and COVID-19 volunteer behavior, along with the moderating role of COVID-19 risk perception in the above relationship. The cross-sectional survey was conducted online using Wenjuanxing from February 12th to March 16th, 2021, in Jiangsu, China. A total of 1,486 participants completed the Toronto Empathy COVID-19 volunteer behavior and COVID-19 risk perception questionnaires. The SPSS PROCESS macro was yielded to examine the moderating effect. Simple slopes analysis was conducted to detect the associations between empathy and COVID-19 volunteer behavior at three levels of the COVID-19 risk perception. The Johnson-Neyman (J-N) technique was used to calculate where the moderating effect is significance. Results showed that empathy was positively related with COVID-19 volunteer behavior (β= 0.080, *p* < 0.001). COVID-19 risk perception played a moderation effect on association between empathy and COVID-19 volunteer behavior (β = −0.005, *p* < 0.001), the greater the levels of COVID-19 risk perception, the weaker the associations between empathy and COVID-19 volunteer behavior. The J-N test showed the association between empathy and COVID-19 volunteer behavior was no longer significant when values of COVID-19 risk perception was >10.71. Current findings could enlighten researchers and policy makers, that fostering volunteerism among public during crisis situation through arousing more empathy and reducing unnecessary risk perception of the public.

## Introduction

Since the COVID-19 outbreak of in late 2019, it has become much more than just a health crisis. Declared a pandemic by the World Health Organization (WHO), COVID-19 has had a heavy impact not just on health, but also on economic recession, joblessness, school closure and social isolation, causing a huge loss of global economy (1). The prevention and control of COVID-19 is an arduous task for all countries as it requires extensive resources, in addition to smart strategies (2). The government undoubtedly played the principal role in leading COVID-19 mitigation measures. However, often, the surge in the emergency requirements during the pandemic have exceeded the capacity of the government and formal organizations (3). In this context, harnessing the power of volunteers together to deal with the COVID-19 crisis has become an important replenishment strategy as volunteers can act as crucial auxiliary human resources helping in residents service, community governance, order maintenance, psychological counseling (4).

The definition of volunteer behavior has been a subject of discussion among scholars, but in a nutshell, it can be conceptualized as any unpaid activity that benefit another person, group or cause (5). Central to this definition is the fact that volunteering generally refers to non-obligatory, unpaid, prosocial, and altruistic behaviors (5–7). Volunteerism is a powerful means of engaging people in tackling disaster response challenges. Volunteerism in emergency and disaster management benefits has affected both communities and the country at large, as it helps to address the shortfalls in funding, resources, and services, and enhances trust, solidarity, and reciprocity among citizens (8–10). Volunteers have been helping significantly during the COVID-19 pandemic in several aspects. For example, in China, after the initial outbreak of COVID-19, volunteers swiftly began helping in the delivery of food, masks, and medicines to people in need; the provision of logistical support for frontline medical staff; and in order maintenance in public places (11). Even after the preliminary containment of the epidemic, tasks related to epidemic prevention and control remain arduous and require supports from volunteers. Therefore, it is necessary to analyze the influence mechanism of volunteering willingness. However, research on volunteering in China remains at a premature stage. This study aimed to analysis the role of empathy on volunteering during COIVD-19, and whether COVID-19 risk perception moderates the relationship between empathy and COVID-19 volunteer behavior.

According to Rodell's review and framework, personality traits greatly influenced one's decision to volunteering (12). Further, empathy was considered to be an essential factor of the personality traits that motivates volunteering (13). Empathy has been conceptualized as a combination of components related to the emotion recognition, comprehension, and responsiveness through sharing the emotional experiences of others, and the perspective through cognitively perceiving others' perspectives (14, 15). Empathy involves cognition and emotions that inspires prosocial behavior and concern for others' welfare (16). Previous studies have shown that empathy is a strong predictor of helping or volunteer behaviors (17–21). The empathy-altruism hypothesis interprets empathy-induced helping behavior, that is, the feelings of concern for others' welfare serve as precursors to altruistic motivation and that feeling empathy toward a person makes them want to help others, regardless of possible personal gains (22, 23). Although empathy has been found to be associated with volunteer behaviors, few studies have explored it in Chinese population, especially during public health emergencies. The traditional Chinese ideology of Confucianism has a strong emphasis on empathy. As written in *Mencius*: “Take care of one's own parents first and extend the same care to the others' parents, take care of one's own children first, and extend the same care to the others' children.” Empathy, one of the most important virtues in Chinese culture, may influence the helping and volunteer behavior of the Chinese public. Thus, we could inference the public's volunteering activities during COVID-19 may have been inspired by their empathy. Thus, we propose Hypothesis 1 (H1): *Empathy has a positive relationship with COVID-19 volunteer behavior*.

Furthermore, risk perception refers to individuals' subjective judgment about the characteristics and severity of a given risk, reflecting one's attitudes or beliefs regarding potential harm (24). In public health emergencies, risk perception is described as individual's feeling and understanding of all kinds of external objective risk, and it emphasizes the influence of intuitive judgment and subjective feeling experience on individual cognition, thus forming a subjective judgment and assessment of the possibility, controllability, and consequence severity of the risk (25). Risk perception is an important factor that influencing individual behaviors. Previous studies found that risk perception influences willingness to engage in volunteer behaviors during a pandemic (26, 27) and helping behaviors during COVID-19 (28). People were exposed to risks of COVID-19 during its epidemic, and there is no doubt that engaging in COVID-19 volunteer activities increases this risk. According to behavioral economic perspective, people prefer to avoid losing compared to gaining the equivalent amount, namely loss aversion (29). Thus, the decision of whether to engaging in volunteer behavior might be made after a tradeoff between the gain (e.g., concerning with others' suffering or needing) and the loss (e.g., their own's safety risk). From this point, when individuals have a high COVID-19 risk perception, they prefer to avoid risk and loss; thus, their willingness to volunteer induced by empathy toward others is weakened. Therefore, we propose Hypothesis 2 (H2): *Risk perception moderates the relationship between empathy and COVID-19 volunteer behavior*.

## Materials and Methods

### Setting and Population

A cross-sectional questionnaire survey was conducted online from February 12th to March 16th, 2021, in Jiangsu, China. Adults over the age of 18 years were invited to participate. Data was collected via Wenjuanxing, a widely used online survey platform in China. The questionnaire was edited online through the platform, and then an electronic two-dimensional code was generated. Participants were recruited through accessing the two-dimensional code in WeChat that sent by researchers and were encouraged to forward the two-dimensional code. A total of 2,065 people accessed the online survey, and 1,503 of them (72.78%) completed the questionnaire. After a data check by the researchers, 17 questionnaires with missing key information or logic errors were excluded. Finally, 1,486 valid questionnaires were included in this analysis (effective response rate was 98.9%).

### Ethical Approval and Informed Consent

This research was approved by the Research Ethics Committee of Nantong University. Due to the anonymity of this study, we could not obtain the participants' written informed consent. However, we make it clear that the survey was voluntary and anonymous at the beginning of the questionnaire. Hence, if a participant completed and successfully submitted his/her questionnaire, their informed consent was assumed.

### Measurement

The questionnaire was divided into two parts. The first part was used to collect socio-demographic information such as sex, age, marital status, educational level, yearly household income and residence. And the second part was used to collect scales or questionnaires information including the participants' empathy, COVID-19 risk perception, and COVID-19 volunteer behavior.

### Empathy

The Toronto Empathy Questionnaire (TEQ) was used to measure empathy. TEQ was a unidimensional measurement of empathy developed by Spreng by synthesizing the marked differences between various multidimensional empathy scales (14). This study used the Chinese short version TEQ-9 revised by Wang with good reliability and validity (30). TEQ-9 consists of nine self-reported items on a Likert-5 scale from 0 (never) to 4 (always). The total score of TEQ-9 range 0 to 36, with higher score indicating higher the empathy. The Chinese short version of TEQ maintained the unidimensional structure of the original questionnaire with good reliability and validity (30). In the present study, confirmatory factor analysis showed that the unidimensional scale had a goodness of fix index (χ^2^/df = 2.07, *p* < 0.001, CFI= 0.866, TLI = 0.821, SRMR=0.065, RMSEA = 0.057), and the Cronbach's alpha for this scale was 0.792.

### COVID-19 Risk Perception

Based on a review of the measurement of risk perception in public health emergencies or pandemics (13), we developed a 12-item COVID-19 risk perception questionnaire comprising four dimensions (severity, uncertainty, uncontrollability, and vulnerability), with each dimension comprising three items. Typified items included “The spread of the COVID-19 pandemic is very extensive” (severity), “It's difficult to accurately predict the tendency to contract COVID-19” (uncertainty), “I think COVID-19 is hard to cure” (uncontrollability), “I am more likely to be infected than others.” (vulnerability). Each question was answered on a Likert-5 scale from 1 (strongly disagree) to 5 (strongly agree). The total score of this questionnaire ranged from 0 to 60, with higher scores reflecting higher COVID-19 risk perception. This questionnaire was tested in pilot survey and showed a good reliability and validity (see Supplementary Tables S1–S3). In the present study, confirmatory factor analysis showed the four-dimensional questionnaire had a goodness of fit index (χ^2^/df = 2.78, *p* < 0.001, CFI = 0.940, TLI = 0.918, SRMR = 0.051, RMSEA = 0.070). The Cronbach's alpha was 0.791 for severity dimension, 0.782 for uncertainty dimension, 0.774 for uncontrollability dimension, and 0.743 for vulnerability dimension, and the Cronbach's alpha of this entire scale was 0.779.

### COVID-19 Volunteer Behavior

COVID-19 volunteer behavior was measured using four items developed by Carlo et al. (31). The items were revised to specifically reflect the context of the COVID-19 pandemic. Three items including “In the past, have you engaged in COVID-19 volunteer activities?”; “Are you currently engaging in COVID-19 volunteer activities?” and “Are you going to participant in COVID-19 volunteer activities in the future?” were answered as yes (=1) or no (=0); and one item of “Are you willing to take part in volunteer activities in the future if needed?” was answered on a Likert-5 scale from 0 (not at all) to 4 (very much). The total score was the sum of the scores of the above items, and a higher score indicated a higher likelihood of taking part in volunteering. This questionnaire was validated by Erez et al. (32) and has been used in the Chinese population (33). The Cronbach's alpha of it was 0.644 in this study.

### Data Analysis

IBM SPSS version 25.0(IBM SPSS Statistics for Windows, IBM Corp., Armonk, NY, United States) was used to conduct the data analyses. Harman's single-factor test was adopted to test the possible common method bias which is a co-variation between the independent and dependent variables derived from the same person in the same measuring using the same questionnaire in self-reported data (34). If the total variation explained by the largest component is below 50%, we could conclude that there is no serious no common method bias in the current study (35).

Participants' socio-demographic characteristics were represented using descriptive statistics. The preliminary bivariate associations of the study variables (empathy, COVID-19 risk perception, and COVID-19 volunteer behavior) were examined using Pearson correlation coefficient.

The moderation effect was analyzed using Model 1 within the SPSS PROCESS macro that developed by Hayes (36). To minimize multicollinearity caused by the independent variable and the product of the independent variable and moderated variable (X^*^W) in moderation regression model (37), the data were centralized before the analysis (38). Socio-demographic characteristics including sex, age, educational level, yearly household income and residence were adjusted for the regression model as they had been correlated with volunteering according to previous study (13). The bootstrapping method in PROCESS macro with 5,000 samples of the data was used to test the robustness of the significant moderating effect, with the results being reported as the effects and their corresponding confidence intervals (CIs). A 95% CI do not contain zero indicated a significant effect (36).

Simple slopes analysis (39) was conducted on the moderating effects to detect relationships between empathy and COVID-19 volunteer behavior at three levels of the COVID-19 risk perception (low means one standard deviation [SD] below the mean; medium means mean, and high means one SD above the mean). The Johnson-Neyman (J-N) technique (40) was used to determine in which value ranges of the moderating variable the interaction (moderating) effect were statistically significant or not (41). J-N technique can analysis the significance regions where simple slope of the dependent variable to the independent variable is significantly different from zero (42). Specifically, when the moderator is continuous variable, J-N test generates point estimation for simple slopes with 95% CIs to test the significance regions of the moderating effect, with a 95% CI do not contain zero indicating significance (43). Thus, J-N test allows us to obtain cutoff points of the moderator at which the effect of the independent variable on the dependent variable is statistically significant (44–46).

## Results

### Participants' Characteristics

There were almost equal numbers of male and female participants (51.5% of female and 48.5% of male). The majority of participants were under 45 years old (84.1%). Nearly half had a junior college degree or higher above (48.0%). Most participants came from urban areas (70.6%), more than twice the proportion from rural areas (29.4%). Furthermore, 54.2% had a yearly household income below 120,000 RMB (Table 1). The age and residence were not fairly distributed with more younger and urban responders include in our study. Such distribution that more younger and urban responders are common in network survey in other published studies (47, 48). In addition, the moderating analysis apply a bootstrapping method with 5,000 samples that is statistically robust against of non-normally or fairly distributed data (49, 50).

**Table 1:** Characteristics of participants (*n* =1486).

**Characteristic**		***n***	**%**
Gender	Male	721	48.5
	Female	765	51.5
Age(years)	<45	1,250	84.1
	≥45	236	15.9
Educational level	vocational school & below	773	52.0
	Junior college & above	713	48.0
Urbanity	Urban area	1,049	70.6
	Rural area	437	29.4
Yearly household income (RMB)	<120,000	805	54.2
	≥120,000	681	45.8

### Testing for Common Method Bias

The result of Harman's single-factor analysis exhibited that six factors with eigenvalues >1 were extracted. The first factor had an explanatory rate of 17.5% of total variances, which was below the recommended threshold of 50%. Thus, we could conclude that the common method bias was not an issue in our study.

### Correlation Analysis

The descriptive statistics and Pearson correlation coefficients for each study variable were presented Table 2. As shown, empathy was significantly positively related with COVID-19 volunteer behavior (*r* = 0.269, *P* < 0.001), while was significantly negatively related with COVID-19 risk perception (*r* = −0.054, *P* = 0.038). In addition, COVID-19 risk perception was significantly negatively related with COVID-19 volunteer behavior (*r* = −0.073, *P* = 0.005). According to Cohen's conventions (51), these results indicated the weak correlations between the study variable (|*r* | <0.3). Given that what the moderating variable needs to affect is the relationship between the independent and dependent variable rather than the dependent or independent variable (41), and the magnitude of correlation coefficient (*r* = 0.269) between the independent and dependent variable is comparable with published studies (52, 53), these results are acceptable.

**Table 2:** Means, standard deviations, and correlations of the major study variables.

	**Variables**	***M***	***SD***	**1**	**2**	**3**
1	Empathy	27.24	5.05	-		
2	Risk perception	39.55	6.20	−0.054^*^	-	
3	Volunteer behavior	4.29	1.53	0.269^***^	−0.073^**^	-

*SD, standard deviations*,

* *p < 0.05*,

** *p < 0.01*,

*** *p < 0.001*.

### Moderating Effect Analysis

The moderating analysis results was presented in Table 3. As shown, empathy was significantly positively associated with COVID-19 volunteer behavior (β = 0.080, *p* < 0.001), thus Hypothesis H1 was thus supported. Meanwhile, the interaction effect of empathy and COVID-19 risk perception on COVID-19 volunteer behavior was significant (β = −0.005, *p* < 0.001). Bootstrapping analysis also showed that the moderating effect of COVID-19 risk perception on the relationship between empathy and COVID-19 volunteer behavior was significant (β= −0.005, Boot 95%CI = [−0.007, −0.003]). Thus, COVID-19 risk perception played a moderating role in the relationship between empathy and COVID-19 volunteer behavior, and H2 was supported. Empathy, COVID-19 risk perception, interaction and control variable accounted for 10.6 % variance in COVID-19 volunteer behavior, with 1.4 % being contributed by the moderating effect (*R*^2^*- Change* = 0.014). These effect sizes were comparable with published studies (54).

**Table 3:** Moderating effect of COVID-19 risk perception on the relationship between empathy and COVID-19 volunteer behavior.

	**β**	**SE**	***t***	**95%CI**
Empathy	0.080	0.007	10.652^***^	0.065, 0.095
COVID-19 risk perception	−0.008	0.006	−1.255	−0.020, 0.004
Interaction	−0.005	0.001	−4.829^***^	−0.007,−0.003
**Control variable**				
Sex	−0.144	0.079	−1.834	−0.298, 0.010
Age	0.089	0.104	0.854	−0.115, 0.293
Educational level	−0.111	0.084	−1.325	−0.277, 0.054
Yearly household income	−0.073	0.082	−0.896	−0.234, 0.087
Urbanity	0.284	0.088	3.216^***^	0.111, 0.458
Constant	4.342	0.078	55.562^***^	4.188, 4.495
*F*	21.903^***^			
*R^2^*	0.106			
*R^2^- Change*	0.014				

** *p < 0.01*,

*** *p < 0.001*.

*Results were shown as centralized scores*.

**Figure 1:**
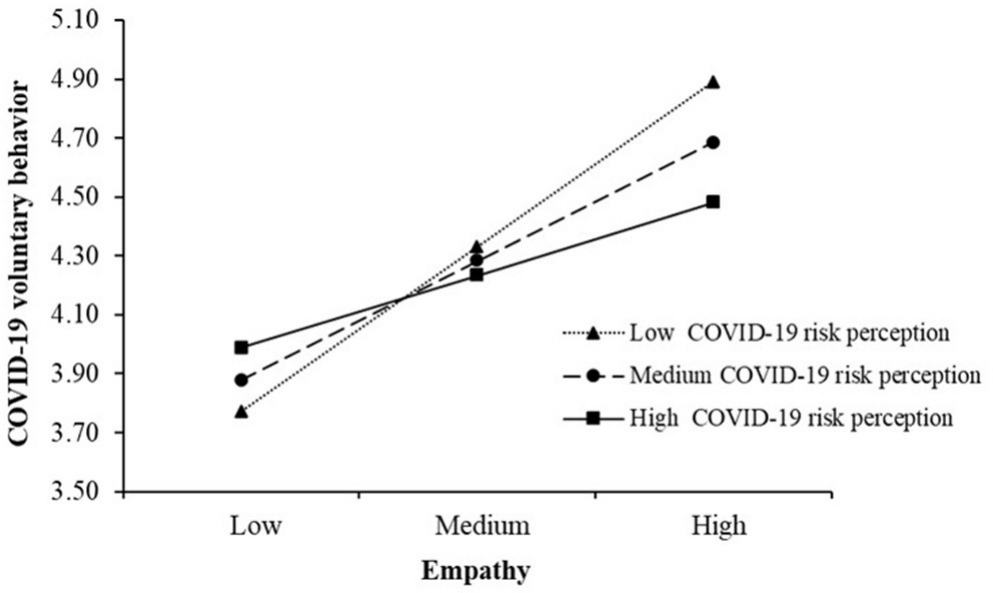
Simple slopes of empathy on COVID-19 volunteer behavior under different levels of COVID-19 risk perception.

As shown in Table 4, simple slopes analysis suggested that the positive association of empathy with COVID-19 volunteer behavior was strongest at low levels of COVID-19 risk perception (β = 0.111, 95%CI = [0.091,0.130]), weaker at medium levels of COVID-19 risk perception (β = 0.080 95%CI = [0.065, 0.095]), and weakest at high levels of COVID-19 risk perception (β = 0.049, 95%CI = [0.029, 0.068]). These results indicated that the associations of empathy with COVID-19 volunteer behavior were weaker in case of higher COVID-19 risk perception. The simple slopes were plotted in Figure 1.

**Table 4:** The effect of empathy on COVID-19 volunteer behavior at different levels of COVID-19 risk perception.

**Levels of COVID-19 risk perception**	**Effect**	***SE***	***LLCI***	***ULCI***
Low (M-1SD)	0.111	0.010	0.091	0.130
Medium (M)	0.080	0.007	0.065	0.094
High (M+1SD)	0.049	0.010	0.029	0.068

*M, mean; SD, standard deviations*.

**Figure 2:**
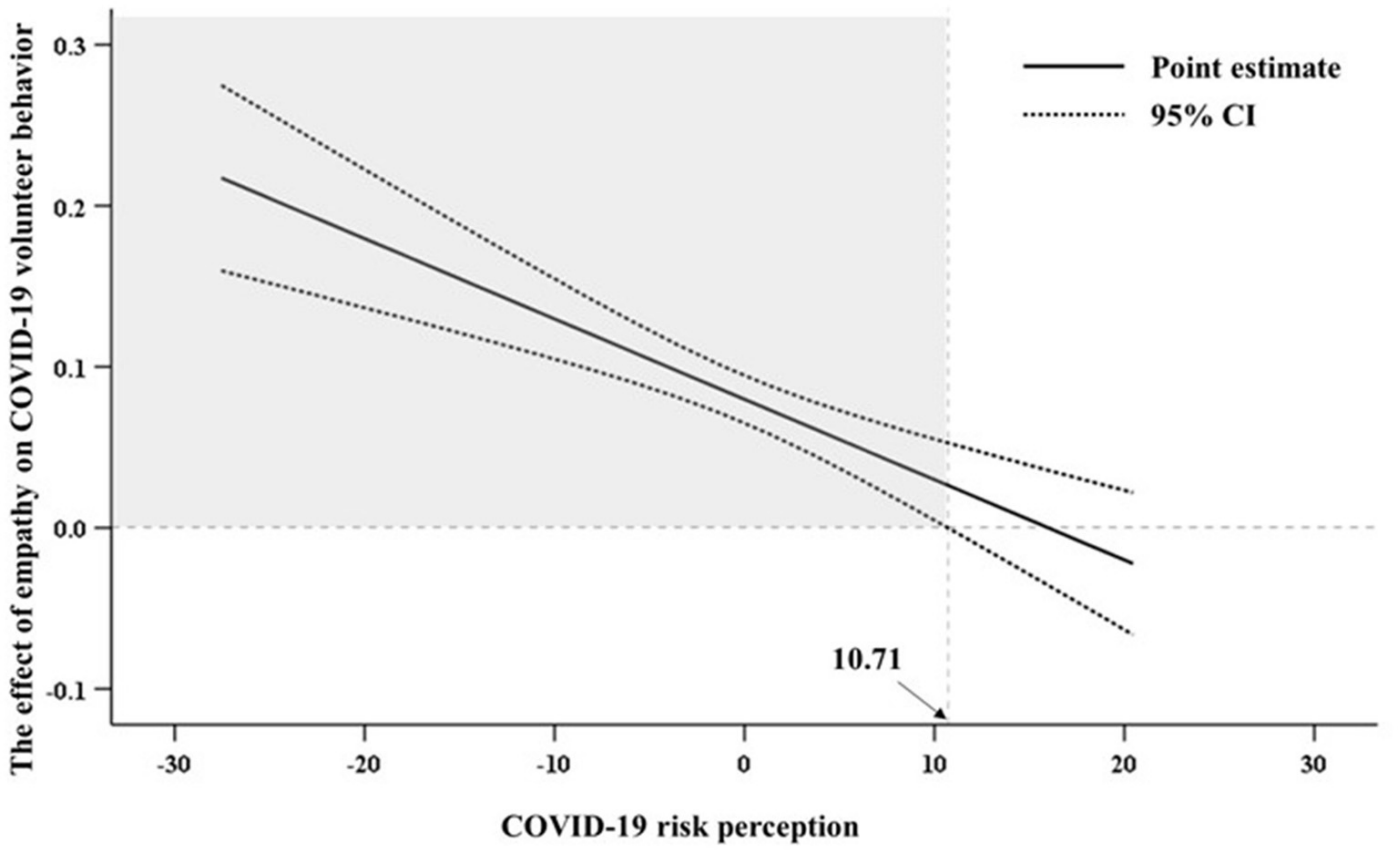
Effect of empathy on COVID-19 volunteer behavior with Johnson-Neyman confidence bands.

The J-N test identified that the association between empathy and COVID-19 volunteer behavior weakened as the values of COVID-19 risk perception increased (Figure 2). Moreover, the above association was significant when the values of COVID-19 risk perception (centralization values) were ≤10.71 (the 95% CIs did not include zero), while was no longer significantly when the values of COVID-19 risk perception were >10.71 (the 95% CIs included zero). Specific results of J-N test could be found in Supplementary Table S4.

## Discussion And Implications

This study found that empathy was positive associated with COVID-19 volunteer behavior, and the associations between empathy and COVID-19 volunteer behavior was moderated by COVID-19 risk perception in such a way that a weaker association between empathy and COVID-19 volunteer behavior was found among those with higher level of COVID-19 risk perception.

Consistent with our Hypothesis H1, we found that empathy had a positive relationship with volunteer behavior, individuals with higher empathy exhibited a higher inclination to serve as volunteers. This finding was accordance with the empathy-altruism hypothesis that claims empathic concern for others in need is an important motivator of helping others through various channels, such as reduction in intergroup conflicts and prejudice and promote of altruism and caring (22, 55). We verified empathy-altruism hypothesis in COVID-19 volunteering among the Chinese public. Empathy is an important component of the psychological prototypes of the Chinese. Like other traditional societies with a long history built on family ties, empathy, defined as “thinking, feeling and imaging one's way inside of others' experience in order to know and understand them,” has been the strongest inner motivation for taking care of a stranger throughout Chinese history (56). Notably, empathy, particularly cognitive empathy, is considered to be an active skill that could be acquired and enhanced through purposeful and informed guidance (57). From this perspective, it is suggested that using social media to broadcast the problems faced by people so as to elicit empathy from viewers and motivate them to participate in volunteering activities to boost volunteer services to compensate for the human resource shortage in the health system during the COVID-19 pandemic. Although empathy serves as a strong antecedent to engage in volunteer behaviors, volunteering is influenced by many other factors including but not limited to personal growth and development, social needs, sense of duty, a sense of purpose and volunteer-work related cognition and attitude (58–60). This is a possible exaptation of small *R*^2^, creating room for other influencing variables. Future research should investigate whether and how these factors relation to volunteer behavior their underlying mechanisms.

Our results also proved that COVID-19 risk perception negatively moderated the relation between empathy and COVID-19 volunteer behavior, supporting our Hypothesis H2. Specifically, the relationship was weaker for individuals with higher COVID-19 risk perception. J-N test also pointed that the association between empathy and COVID-19 volunteer behavior weakened as the values of COVID-19 risk perception increased, and such association was no longer significant when the values of COVID-19 risk perception was >10.71 (centralization value). From behavioral economics perspective, in the face of rapid transmission of COVID-19, difficult control, and unclear transmission mechanisms, individuals with a higher level of COVID-19 risk perception might pay more attention to their risk loss (such as avoiding the risk to their own's and their family members) rather than their interests (such as concerning with other's needing). Thus, the higher the COVID-19 risk perception, the lower the empathy-induced volunteer behavior. Although the moderation effect is small, it is still significant. The one is that a small effect size could have significant theoretical implications if it could support the theoretical hypothesis (61). The other is that a small effect size also could have realistic implications if it could lead to important outcomes such as volunteer behavior during COVID-19 emergency or if it involves relatively wide population such as in China with a large population (61).

Risk perception is important during COVID-19 epidemic as it influence people's behavioral response, such as the adherence to infection control measures (62, 63). Up to now, China is taking the strictest prevention and control measure by adhering to the dynamic zero-COVID policy, such as quarantine, management, lockdown, and screening. The Chinese public and society maintain high risk awareness of COVID-19 in daily life. Given that higher risk perceptions have a negative impact on helping and volunteer behavior, policymakers should pay much attention to risk-ensuring system for emergency volunteers to motivate volunteering engagement (64). Meanwhile, the risk perception variable is usually decomposed into different dimensions (47), future research that investigates each dimension and its effect on volunteer behavior could be able to provide more useful indications to policymakers.

It was demonstrated that public perception of risk is related to information dissemination by the media risk communication (65). Extensive and intensive reports on emergencies may lead to high perception of risk. Despite the negative effect of risk perception on the efficiency of empathy evoking volunteer behavior, we do not suggest any authority who has the power to simply shut the public from risk information during crisis in order to foster volunteerism. This is not only because telling half the truth is unethical and probably illegal, but also it may also reduce people's trust in the government. Previous studies have shown that if the information is provided in a matched manner, it may have considerable benefits in reducing possible misunderstandings or biases among the public (66). Although focusing on ones' own risks and benefits could impede prosocial behaviors, these adverse effects can be offset by increasing information about the benefits of prosocial behaviors by emphasizing the high risks faced by others (67). As explained previously, understanding the victim's suffering is essential in evoking individuals' empathy. Only by seeing the full picture of the real situation can volunteers try to help others in need and take care of themselves. Therefore, optimal risk information communication strategies that considering multiple elements are imperative, aiming at reducing the public's risk perception without misleading risk information during a crisis instead of shutting the public away from risk information (68, 69).

Findings of the current study also revealed that men tended to have a higher COVID-19 risk perception compared to women, which was in contrast to some other studies, but in line with one study of the COVID-19 (66). This result can be explained by the higher mortality rate for men that is highly reported in the media (70). It is worth mentioning that the public from rural regions had a higher willingness to engage in COVID-19 volunteer behavior than those from urban regions. This difference is caused by the fact that the public from rural regions had a higher empathy level and a lower COVID-19 risk perception level.

Volunteers have been proven to be helpful in several aspects during COVID-19 outbreak and the regular COVID-19 control, especially in terms of compensating for the shortage of human resources (33). China has not established a sound system to protect volunteers' interests and health during the activities of devoting themselves to help others, which make volunteers more vulnerable as they put themselves into the teeth of the storm. Therefore, a system to take care of volunteers ahead of fostering volunteerism needs to be constructed as urgently as possible against the background that COVID-19 is expected to be the “new normal” for a long time. This system should consider the cultivation of volunteers, response protocols in different situations, working standards, and a strong volunteers security system (71).

### Limitations

Several limitations should be acknowledged in this study. First, this survey was conducted through online survey, although it could reach a large number of responders under the social distancing regulations in China, the non-random sampling method might lead to sample bias. Second, this study was only conducted in one province, so the generalizability of our findings needs to be cautious. Third, this was a cross-sectional study, therefore, it prevented us from establishing causal relationships between the study variables. Longitudinal data is needed as exogenous variables or external factors have changed, such as the emotion, attitude and risk perception, the disease epidemic of COVID-19 disease epidemic, and the volunteer service management system so that their relationship with volunteerism and their underlying mechanisms could be explored. Fourth, the data were self-reported, therefore, recall bias and social desirability bias might exist.

## Conclusion

This study indicated that empathy had a positive association with COVID-19 volunteer behavior. Moreover, COVID-19 risk perception played a moderating role in the relationship between empathy and COVID-19 volunteer behavior in the way that weakened the relationship between empathy and COVID-19 volunteer behavior. To foster more volunteer service behavior among public during social crisis, proper risk information strategies to reduce the unnecessary public risk perception as well as to improve empathy guided a more comprehensive understanding of what is really happening are suggested. Policymakers and non-governmental organizations should contribute jointly to building a sustainable incentive volunteer system to encourage the public to engage in volunteer behaviors for the well-being of society and to protect volunteers' own interests.

## Data Availability Statement

The data supported this study could be available from the corresponding author with reasonable request.

## Author Contributions

YZ and MZ contributed to conception and design of the study. MZ organized the database. YZ performed the statistical analysis and wrote the first draft of the manuscript. JZ wrote sections of the manuscript. BL, HL, and JR reviewed and revised the original manuscript. All authors contributed to manuscript revision, read, and approved the submitted version.

## Funding

The research was funded by the National Natural Science Foundation of China (72004104), the Social Science Foundation of Nantong (2020CNT010), the Project of Philosophy and Social Science Planning in 2019–2020 in Zhuhai (2019ZC106), the Public Welfare Project of Science and Technology Department in Zhejiang (LGF22G030018), and the Post-Doctoral Fund of Heilongjiang Province (LBH-Z21181).

## Conflict of Interest

The authors declare that the research was conducted in the absence of any commercial or financial relationships that could be construed as a potential conflict of interest.

## Publisher's Note

All claims expressed in this article are solely those of the authors and do not necessarily represent those of their affiliated organizations, or those of the publisher, the editors and the reviewers. Any product that may be evaluated in this article, or claim that may be made by its manufacturer, is not guaranteed or endorsed by the publisher.
