# Supplementary material for: The Moderating Effect of COVID-19 Risk Perception on the Relationship Between Empathy and COVID-19 Volunteer Behavior: A Cross-Sectional Study in Jiangsu, China
Source: Front Public Health. 2022 Jun 16;10:863613. doi: 10.3389/fpubh.2022.863613 (PMC9243539; doi:10.3389/fpubh.2022.863613)
Supplement: Supplementary file 1 [file Table_1.DOCX]

Supplementary Material

**Table S1.** Cronbach’s alpha of COVID-19 risk perception questionnaire in pilot survey^*^.

|  | Cronbach’s α if item is dropped^**^ |
| --- | --- |
| Item1 (The spread of the COVID-19 pandemic is very extensive) | 0.830 |
| Item2 (The health impact of COVID-19 epidemic is very serious) | 0.828 |
| Item3 (The COVID-19 pandemic will have a negative impact on society) | 0.829 |
| Item4 (The impact of COVID-19 epidemic may surpass previous epidemics) | 0.827 |
| Item5 (It’s difficult to accurately predict the tendency to contract COVID-19) | 0.832 |
| Item6 (The source and transmission mechanism of COVID-19 are still unclear) | 0.832 |
| Item7 (The COVID-19 is hard to cure) | 0.831 |
| Item8 (The spread of COVID-19 is difficult to control) | 0.836 |
| Item9 (The prevention and control role of COVID-19 vaccination is limited) | 0.840 |
| Item10 (If COVID-19 continues, I have a high chance of contracting it) | 0.838 |
| Item11 (I am more likely to be infected with COVID-19 than others) | 0.826 |
| Item12 (Once I contact with novel coronavirus or COVID-19 patient, I will be infected) | 0.841 |
| Overall Cronbach’s α of the questionnaire was 0.844 |  |

**Note:** ^*^The pilot survey included 97 participants. ^**^Cronbach’s α do not change greatly if one item is deleted indicated a good internal consistency of the 12 items and the overall questionnaire.

**Table S2.** Exploratory factor analysis of COVID-19 risk perception questionnaire in pilot survey^*^.

|  | Factor | | | | | |
| --- | --- | --- | --- | --- | --- | --- |
|  | 1 | 2 | 3 | | 4 | |
| Item1 |  |  | |  | | 0.418 |
| Item2 |  |  | |  | | 0.953 |
| Item3 |  |  | |  | | 0.483 |
| Item4 | 0.699 |  | |  | |  |
| Item5 | 0.833 |  | |  | |  |
| Item6 | 0.633 |  | |  | |  |
| Item7 |  | 0.655 | |  | |  |
| Item8 |  | 0.691 | |  | |  |
| Item9 |  | 0.684 | |  | |  |
| Item10 |  |  | | 0.701 | |  |
| Item11 |  |  | | 0.692 | |  |
| Item12 |  |  | | 0.650 | |  |
| KMO | 0.752 |  | |  | |  |
| Bartlett χ^2^ | 438 |  | |  | |  |
| *P* | <0.001 |  | |  | |  |

**Note:** ^*^The pilot survey included 97 participants. Factors were extracted by maximum likelihood method and rotated by varimax rotation. Factor 1 was uncertainty. Factor 2 was uncontrollability. Factor 3 was vulnerability. Factor 4 was severity.

**Table S3.** Fit indexes in confirmatory factor analysis of COVID-19 risk perception in pilot survey^*^.

| **χ^2^** | ***df*** | ***P*** | **CFI** | **TLI** | **SRMR** | **RMSEA** |
| --- | --- | --- | --- | --- | --- | --- |
| 77.3 | 48 | 0.005 | 0.927 | 0.899 | 0.055 | 0.079 |

**Note:** ^*^The pilot survey included 97 participants.

**Table 4.** The J-N test for defining significance region of the moderating effect at values of COVID-19 risk perception.

| **Values of COVID-19**  **risk perception** | **Effect** | **SE** | **t** | ***p*** | ***LLCI*** | ***ULCI*** |
| --- | --- | --- | --- | --- | --- | --- |
| -27.550 | 0.217 | 0.029 | 7.404 | 0.000 | 0.160 | 0.275 |
| -25.150 | 0.205 | 0.027 | 7.616 | 0.000 | 0.152 | 0.258 |
| -22.750 | 0.193 | 0.025 | 7.864 | 0.000 | 0.145 | 0.242 |
| -20.350 | 0.181 | 0.022 | 8.156 | 0.000 | 0.138 | 0.225 |
| -17.950 | 0.169 | 0.020 | 8.504 | 0.000 | 0.130 | 0.208 |
| -15.550 | 0.157 | 0.018 | 8.920 | 0.000 | 0.123 | 0.192 |
| -13.150 | 0.145 | 0.015 | 9.421 | 0.000 | 0.115 | 0.176 |
| -10.750 | 0.133 | 0.013 | 10.017 | 0.000 | 0.107 | 0.160 |
| -8.350 | 0.121 | 0.011 | 10.696 | 0.000 | 0.099 | 0.144 |
| -5.950 | 0.109 | 0.010 | 11.366 | 0.000 | 0.091 | 0.128 |
| -3.550 | 0.098 | 0.008 | 11.747 | 0.000 | 0.081 | 0.114 |
| -1.150 | 0.086 | 0.008 | 11.298 | 0.000 | 0.071 | 0.100 |
| 1.250 | 0.074 | 0.008 | 9.656 | 0.000 | 0.059 | 0.089 |
| 3.650 | 0.062 | 0.008 | 7.307 | 0.000 | 0.045 | 0.078 |
| 6.050 | 0.050 | 0.010 | 5.054 | 0.000 | 0.030 | 0.069 |
| 8.450 | 0.038 | 0.012 | 3.252 | 0.001 | 0.015 | 0.060 |
| 10.711 | 0.026 | 0.013 | 1.962 | 0.050 | 0.000 | 0.053 |
| 10.850 | 0.026 | 0.014 | 1.893 | 0.059 | -0.001 | 0.052 |
| 13.250 | 0.014 | 0.016 | 0.873 | 0.383 | -0.017 | 0.044 |
| 15.650 | 0.002 | 0.018 | 0.096 | 0.924 | -0.033 | 0.037 |
| 18.050 | -0.010 | 0.020 | -0.508 | 0.611 | -0.050 | 0.029 |
| 20.450 | -0.022 | 0.023 | -0.988 | 0.323 | -0.066 | 0.022 |

Note: The association between empathy and COVID-19 volunteer behavior was no longer significant when values of COVID-19 risk perception greater than 10.71, results are shown as centralization values.
